# Effects of Physical Exercise on Cardiometabolic Health in Individuals with Autism Spectrum Disorder: A Systematic Review

**DOI:** 10.3390/healthcare13040439

**Published:** 2025-02-18

**Authors:** Léa Charlier, Léa Cordeiro, Jorge Lopes Cavalcante Neto, Étore De Favari Signini, Jordana Barbosa-Silva, Camilo Corbellini, Antoine Lipka, Raphael Martins de Abreu

**Affiliations:** 1 Department of Health, LUNEX University of Applied Sciences, 4671 Differdange, Luxembourg; charlier.lea@stud.lunex.lu (L.C.); cordeiro.lea@stud.lunex.lu (L.C.); ccorbellini@lunex.lu (C.C.); lipka.antoine@stud.lunex.lu (A.L.); 2 Department of Humanities Sciences, University of Bahia State, Jacobina 40411120, Brazil; jorgelcneto@hotmail.com; 3 Department of Physiotherapy, Federal University of São Carlos, São Carlos 13565905, Brazil; efsignini@estudante.ufscar.br; 4 Faculty of Business Management and Social Sciences, University of Applied Sciences—Hochschule Osnabrück, 49076 Osnabrück, Germany; j.barbosa-da-silva@hs-osnabrueck.de; 5 Department of Health, LUNEX ASBL Luxembourg Health & Sport Sciences Research Institute, Luxembourg LUNEX University of Applied Sciences, 4671 Differdange, Luxembourg

**Keywords:** autism spectrum disorder, exercise, cardiorespiratory fitness, cardiovascular health

## Abstract

Background/Objectives: Individuals with autism spectrum disorder (ASD) are at increased risk of developing cardiometabolic diseases. Although physical exercise (PE) has emerged in the literature as an important modulator for reducing such risk, evidence remains unclear. This systematic review aimed to investigate the effects of PE on cardiometabolic health in individuals with ASD. Methods: A systematic review was carried out according to the PRISMA guidelines, from their inception until 18 July 2023, in the following electronic databases: Scopus, Medline, and Web of Science. Studies were included if they focused on ASD patients undergoing physical exercise, assessing cardiometabolic risk, exercise tolerance, and QoL. The following were excluded: non-exercise interventions, additional therapies, non-English studies, and reviews. The methodological quality of the included studies was assessed through the Downs and Black scale. Results: A total of four studies (149 participants) were included in this review, with the average methodological quality being rated as “fair”. Interventions had mixed effects on cardiometabolic health. The BMI (↓2.8 kg/m^2^), waist circumference (↓1.86 cm), and lipid profiles improved in some cases. VO_2_max and HR_baseline_ showed moderate gains. Secondary outcomes included enhanced endurance, strength, and calorie expenditure, especially in mild ASD. Autistic traits and quality of life improved post-intervention, with better results in the experimental groups. Conclusions: This review indicates that aerobic and functional training improves cardiometabolic health, autistic traits, and QoL in individuals with ASD, particularly in mild cases. Further research is needed to explore the impact of ASD severity on these outcomes.

## 1. Introduction

Autism spectrum disorder (ASD) involves lifetime difficulties with repetitive behavior and restricted interest and social communication. Approximately 1/100 children are diagnosed with ASD worldwide [1]. There has been a high increase in ASD prevalence since 2010 in studies from several countries. Although the etiology of ASD is still unclear in the literature, spatiotemporal disturbances in brainstem development spreading toward the cerebral cortex could be a part of an explanation [2].

Low levels of physical activity in individuals with ASD can be associated with obesity and overweight [3], which can increase arterial stiffness in the population with ASD [4] and the risk of cardiovascular events. People with ASD have an increased risk of having cardiometabolic diseases. They are 1.57 times more likely to develop diabetes overall (1.64 times for type 1 and 2.47 times for type 2), 1.69 times more likely to get dyslipidemia, and 1.46 times more likely to develop heart disease [5,6]. In addition, people with ASD have higher triglyceride concentrations [6]. Moreover, heart rate variability (HRV) [7] and cardiorespiratory fitness (VO_2_peak) [8] are lower in people with ASD when compared with typically developed children. Finally, people with ASD have lower energy capacity and higher energy expenditure [9], which might be linked to the abovementioned changes.

Furthermore, individuals with ASD often engage in limited physical exercise (PE) [10]. This reduced activity may be attributed to interpersonal challenges that impede their involvement in regular physical activities. Factors such as misunderstandings and disagreements with family or friends can be discouraging, leading to decreased participation [11]. Additionally, difficulties in social skills further compound this issue [10]. Considering these challenges, it is noteworthy that PE holds significant promise for individuals with ASD. A previous study suggests that engaging in physical activities can positively impact social and communication skills among patients with ASD [12]. Moreover, it improves muscular strength, endurance, manipulative skills, locomotor skills, and skill-related fitness, ultimately enhancing exercise capacity [13]. Despite the recognized benefits of PE, there is a gap in the current guidelines, as outlined by the American Heart Association [14]. The recommended practice of 150 min per week of moderate-intensity aerobic activity does not account for the unique needs of patients with ASD, given that not all conventional exercises may be suitable for them. This underscores the importance of tailoring exercise interventions to address the specific challenges faced by individuals with ASD.

Therefore, this systematic review aims to investigate the effects of PE on cardiometabolic health in individuals with ASD. To our knowledge, no systematic review or literature review has investigated the impact of PE on the cardiometabolic health of people with ASD, which could be helpful in effectively guiding sport and rehabilitation professionals in exercise prescription for this population. We hypothesized that similar to neurotypical people [15], participants with ASD will improve their cardiometabolic health through PE.

## 2. Materials and Methods

### 2.1. Database and Search Strategy

This systematic review was carried out according to the PRISMA guidelines [16]. This study included research from the guidelines’ inception until 18 July 2023, based on the following databases: SCOPUS, Medline, and Web of Science. The following terms were used and combined in the electronic databases: (exercise OR physical activity OR aerobic exercise OR resistance training OR strength training OR fitness OR sports) AND (cardiovascular health OR metabolic health OR cardiorespiratory fitness OR lipid profile OR blood pressure OR blood glucose OR insulin resistance OR obesity) AND (autism OR autistic disorder OR Asperger syndrome OR pervasive developmental disorders OR ASD). After conducting the electronic database search, the results were imported into Rayyan, a specialized electronic tool for systematic reviews (https://rayyan.qcri.org (accessed on 18 July 2023)). Moreover, duplicate documents were identified and removed by using the duplicate function in the application. Only studies in English were retained. No restriction about the publication date was considered. Two independent researchers (L.C. and L.C.) proceeded to screen the title and abstract following the eligibility criteria. Then, a meeting was conducted to obtain a consensus on the selected articles. A third researcher (R.M.A.) was consulted in case of any disagreement. The same procedure was replicated to screen the full text and perform data extraction and quality assessment. The systematic review protocol was registered in the PROSPERO database under the following protocol number: CRD42023441899.

### 2.2. Study Criteria and Eligibility

The eligibility criteria are based on the Participant Intervention Comparison Outcome (PICO) format. Only primary studies were included if they focused on individuals diagnosed with ASD at any level of severity (P) receiving any modality of PE (I) compared with no PE/standardized care (C). The outcome (O) of interest was cardiometabolic risk factors. Exercise tolerance and quality of life (QoL) were secondary outcomes. On the other hand, studies focused on multiple diseases, studies in which participants received additional therapy that could influence the outcomes, interventions that do not include physical exercises, non-English studies, conference papers, books and letters, case studies, thesis, guidelines, qualitative studies with absence of numerical data, systematic reviews, and meta-analyses were excluded from this review. Thus, studies were not retained if they failed to report quantitative data or were not primary studies (i.e., systematic reviews or meta-analyses).

### 2.3. Quality Assessment

The Downs and Black checklist was used to analyze the quality of the retrieved studies. It consisted of 27 items gathered in five subcategories: (1) Reporting (10 items), “which assessed whether the information provided in the paper was sufficient to allow a reader to make an unbiased assessment of the findings of the study”; (2) External validity (3 items), “which addressed the extent to which the findings from the study could be generalized to the population from which the study subjects were derived”; (3) Bias (7 items), “which addressed biases in the measurement of the intervention and the outcome”; (4) Confounding (6 items), “which addressed bias in the selection of study subjects”; (5) Power (1 item), “which attempted to assess whether the negative findings from a study could be due to chance”. Each item scored either 0 (“unable to determine” or “no”) or 1 (“yes”), except for 1 item in the Reporting subcategory, which scored 0 (“no”), 1 (“partially”), or 2 (“yes”), and the item on power, scored from 0 to 5 [17]. Initially, the checklist was scored out of 32 points. Several authors have used a modified version of the checklist that simplifies the scoring of item 27 as 1 if there is “sufficient power to detect a clinically important effect, where the probability value for a difference due to chance is <5%” [18]. This allowed us to have a maximum total score of 28 points and use the following quality of evidence: excellent (26–28), good (20–25), fair (15–19), and poor (≤14) [19].

### 2.4. Data Extraction

From each selected study, the characteristics of the study and participants (i.e., authors, groups, age, gender of participants, and primary outcomes values at baseline) were extracted in the first table. The primary outcomes of interest were risk factors of cardiometabolic health like weight, body mass index (BMI), cholesterol (total, HDL-C, and LDL-C), glucose levels, heart rate (HR), waist circumference (WC), allometric index (WHT5), and VO_2_max. The characteristics of each intervention (i.e., type of exercise, duration of intervention, frequency, intensity, duration of each training session, and control group intervention) and the post-intervention effects on the primary outcomes and secondary outcomes (i.e., assessment of QoL and exercise response values such as Child Health questionnaire-CHQ-PF50, calorie expenditure, fat mass, muscle endurance, strength, and basal metabolic rate) were gathered in two other tables.

### 2.5. Primary Outcomes

Cardiometabolic disorders are “a cluster of conditions including abdominal obesity, insulin—resistant glucose metabolism, dyslipidemia, and increased blood pressure” [20]. This study focused on modifiable risk factors, such as total cholesterol, HDL-C, LDL-C, HR, glucose levels, BMI, and triglycerides, which are considered relevant by several algorithms for quantifying cardiometabolic risks. Non-traditional risk factors such as metabolic syndrome are also important [21]. Abdominal obesity, defined by waist circumference, is relevant for properly assessing cardiometabolic disease risk. Some consider the WHT5 efficient in screening cardiometabolic risk [22]. Cardiorespiratory fitness based on VO_2_max is an important indicator of cardiovascular and metabolic risk in adults and young individuals [23].

### 2.6. Secondary Outcomes

A study has proven that high caloric expenditure during an effort is significantly more effective than regular cardiac rehabilitation for weight loss and improves cardiometabolic risk factors in patients with coronary disease [24]. Basal metabolic rate, calorie expenditure, muscle endurance, muscle strength, cardiopulmonary endurance (shuttle run test), and fat mass will be reported about this population’s late exercise response and tolerance. Social and psychological situations are primordial non-traditional risk factors for cardiovascular disease in people with ASD [21]. Adults with ASD are at higher risk of mental health problems like depression, obsessive–compulsive disorder, anxiety, and attention deficit hyperactivity disorder [25]. Psychosocial risk factors were extracted considering the Child Health questionnaire-CHQ-PF50.

## 3. Results

### 3.1. Study Selection

**Figure 1:**
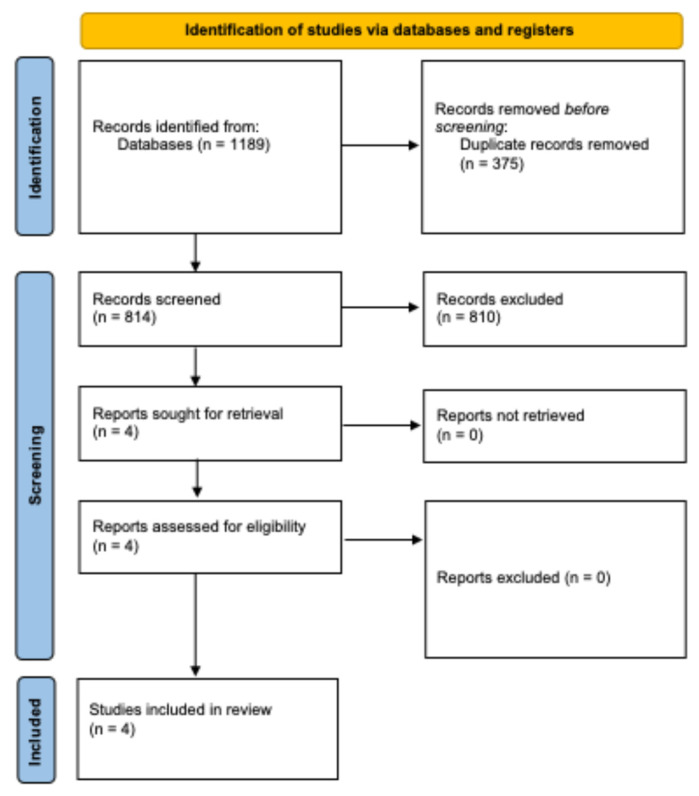
PRISMA flow chart of search strategy and retrieval of articles [26].

Following the search strategy, 1189 documents were found in the electronic databases (PubMed = 308, Scopus = 389, and Web of Science = 492). Three hundred seventy-five duplicates were deleted. After screening the title and abstract, eight hundred ten articles were excluded because they did not meet the eligibility criteria. Four articles were included in this systematic review, and no disagreements between the independent reviewers were found (Figure 1). The main reasons for exclusion by title and abstract were (a) multiple diseases; (b) non-cardiometabolic outcomes as primary outcomes; (c) nonphysical exercise therapy, therapy outside of the physiotherapist’s scope of practice, or multiple interventions; and (d) metanalyses, reviews, case studies, guidelines, theses, dissertations, qualitative studies, abstracts, acute interventions, and non-English language articles.

### 3.2. Characteristics of Participants and Groups

Table 1 describes the characteristics of the participants and groups. The total sample size was 149 participants aged from 6 to 29 years. Three studies described the male/female ratio: 97 males compared to 12 females were reported [27,28,29]. One study did not specify the genders of their participants [30]. The number of individuals per group ranged from 5 to 46. Two studies reported the level of severity of the participants’ condition: one assessed a person with severe ASD [28], while the other focused on participants with mild ASD and ones with severe ASD [27].

### 3.3. Characteristics of Interventions

The interventions are reported in Table 2. Three studies compared PE with leisure activity [28] or daily level of activities [29,30]. One study assessed the effects of exercises on two groups of participants: those with mild ASD and those with severe ASD [27]. The exercise type that the experimental group (EG) executed differed. One was endurance training [28], two interventions were coordination and strength exercises [27,29], and the last one was a judo-like program [30]. The intensities of effort were described in diverse ways: one study used speed to determine the intensity (mild to moderate intensity), from 2.4 mph to 4.1 mph [28]. Two studies used the type of movement and duration of each task to determine the intensity of each exercise [27,30]. Finally, one study determined the exercise load by describing the task and using weight, number of sets, and time [29]. No biological markers or specific scales were used to determine the intensity of the effort. The intervention time ranged from 8 [28] to 90 min per session [30] for 12 weeks [27] to 48 weeks [29]. The frequency was 2 times a week for three studies [27,28,29] and once a week for one study [30]. The intervention was performed with instructors with or without experience working with individuals with ASD [27,29,30] and guidance staff members [28].

The measurement methods of each outcome of interest were quite similar in the two studies. Two studies reported the number of observers measuring anthropometric values: one observer [29] and two observers [30]. The other two studies did not specify the number of observers. Two procedures assessed the participant’s height and weight: two opted for stadiometers and balances [29,30], while the other used bioelectrical impedance analysis with the IOI353 Model [27]. The study participants had to place themselves on foot electrodes before and after voiding their dinner, and the machine analyzed their composition and segmental impedance. To calculate the BMI of the participants, all studies used the equation BMI= weight/height squared [28,29,30]. Finally, the two studies that measured the WC of their participants used non-elastic tapes that they placed above the iliac crest with the feet together and abdomen relaxed and midway between the iliac crest and ribs, near the umbilicus, feet–shoulder width, with the abdomen relaxed [29,30]. Then, one study used the formula WHT.5R = WC/height0.5 to obtain the participants’ values of the allometric index [30]. The biochemical variables were collected through blood samples pre- and post-intervention, after 12 h of overnight fasting. The authors used Fiedewald’s formula to calculate LDL-C and the enzymatic method to measure glucose [29]. Concerning cardiac health, two studies tried to assess the HR of their participants: one failed to measure it because the participants lacked cooperation [28] and made a family member of the participants measure HR_baseline_ with a finger pulse oximeter for 1 min for four days right after waking up, before and after the intervention [30]. To finish, they also estimated the VO_2_max of their participants by using a non-exercise equation based on HR_baseline_, WC, age, and intensity of weekly activity (obtained with the physical activity index) [30]. In another study, the calorie expenditure of their participants was defined with an equation of the American College of Sports Medicine: METS × 3.5 × body weight in kg)/(200) (with METS = net VO_2_/3.5 mL kg^−1^ min^−1^ and net VO_2_ = 0.1 (speed of treadmill) + 1.8 (speed of treadmill) (fractional grade of treadmill) + 3.5) [28].

### 3.4. Results of Primary Outcomes

Table 3 summarizes the effect of each intervention on cardiometabolic health outcomes. The results are controversial. Most of the studies reported no significant changes in weight [28,29]. The BMI decreased, in most studies, by up to −2.8 kg/m^2^ [27,28]. WC underwent different changes: while one study showed no significant changes, the other showed an improvement in WC of up to −1.86 cm [30]. The WHT5 was improved by 0.024 [30]. Only one study compared cholesterol, glucose, and triglycerides pre- and post-intervention [29]. In the EG, there was a significant decrease in total cholesterol (−10.1 mg/dL) and LDL-C (−7.7 mg/dL), a significant increase in HDL-C (+5.2 mg/dL), and no significant changes in triglycerides and glucose levels. HR_baseline_ and VO_2_max were assessed only by one study [30]: the EG decreased its HR_baseline_ with a medium effect size and improved its VO_2_max by a medium increase. Overall, the control groups (CGs) of the four studies went through no significative changes or worsened their cardiometabolic health outcomes, except for the WHT5 and triglyceride levels (−0.008 and −38.1 mg/dL, respectively) [29,30].

### 3.5. Methodological Quality Assessment

The details of each methodological quality assessment of the studies are described in Table 4. The studies had average Downs and Black scale scores of 16.5/28, corresponding to fair methodology quality. The main lack of information was about (a) the recruitment of the participants, (b) the proportion of confounding factors in the sample, and (c) the period of recruitment. In addition, the studies lacked (d) the randomization of both healthcare staff and participants and (e) the consideration of the confounding factors during the analysis of the main findings. None of the studies were excluded due poor or fair quality.

### 3.6. Results of Secondary Outcomes

One of the studies used the shuttle run test to assess cardiopulmonary endurance. Participants with mild ASD improved their shuttle run test by ~8.49% and their fat mass by ~0.28% and increased their BMR by ~0.75%, their muscle endurance by ~11.34%, and their strength by ~16.92% [27]. Participants with severe ASD worsened their results of the shuttle run test by ~2.88%; decreased their basal metabolic rate and muscle endurance by ~1.39% and ~12.48%, respectively; and increased their strength and their fat mass by ~17.20% and ~5.60%, respectively. One study assessed the effect of PE on calorie expenditure during treadmill run [28]. The EG improved their calorie expenditure between month 1 (168.38 ± 74.1) and month 9 (914.62 ± 233.4) of intervention. Another study reported changes in autistic traits and QoL after intervention [29]. The autistic traits scale score decreased for both groups after the intervention (CG: −1.4; EG: −8.1). The EG improved their physical and psychosocial health (13.3 and 15.2, respectively). Physical health improved slightly for the CG (3.1), but their psychosocial health decreased (−2.4). Finally, the results of the motor profile scale increased almost not significantly for the CG (0.1) and decreased for the EG (−2.4).

## 4. Discussion

This systematic review aimed to investigate the effects of PE on cardiometabolic health in individuals with ASD. Our search strategy resulted in a total of four articles that considered the effects of PE on cardiometabolic outcomes. Two studies identified no weight change after a PE protocol, although these results are debatable for the BMI [28,29]. While another study reported no significant changes [29], one of the studies had opposite results depending on the severity of ASD, i.e., the population with mild ASD improved their weight and BMI [27]. The ones with severe ASD worsened those outcomes contrary to the participants of one study [28], where the BMI was significantly improved. Additionally, WC was improved in one study [30] but did not change for participants in another [29]. The WHT5 decreased after intervention [30]. An improvement in cholesterol levels and no change in glucose levels and triglyceride levels were also reported [29]. After the intervention, both HR_baseline_ and VO_2_max improved [30]. All secondary outcomes improved [27,28,29] except the fat mass, basal metabolic rate, and cardiorespiratory and muscle endurance in the population with severe ASD [27]. Therefore, PE demonstrates potential for improving key cardiometabolic outcomes in individuals with ASD, particularly in enhancing cardiovascular fitness and cholesterol levels. These findings suggest that exercise can be a valuable component of health interventions for this population. While the variability in the results points to the need for more research, the positive effects observed highlight the importance of incorporating PE into practice for professionals working with individuals with ASD, offering benefits that can support overall health and well-being.

### 4.1. Primary Outcomes

The literature reports that especially in overweight populations, exercise improves body composition [31,32] but does not have long-term effects on weight [31], which correlates with our results. Fat loss is more important in subcutaneous adipose tissue (SCAT), since it is the area with the largest fat depot, but the relative change is greater in visceral adipose tissue (VAT) [33]. One study explains that exercise induces SCAT adaptation by increasing mitochondrial activity and altering lipid metabolism [34]. Men lose SCAT mostly from the abdomen [33], which corroborates the changes in WC and the WHT5 reported previously. The formula of the BMI, which is weight/height^2^, might explain the more substantial and statistically significant changes. Indeed, despite the absence of significant weight decrease, one study reported an improvement in the BMI [28]. These findings are important, as a higher BMI has been associated with increased aortic blood pressure and increased aortic stiffness for children with ASD [6]. Stiffer aortas are associated with smaller brain volumes and increase the risk of cardiovascular diseases and cognitive impairment [35]. Moreover, exercise-induced VAT reduction decreases the risk of cardiovascular and metabolic diseases and mortality [36].

Different exercise-induced mechanisms of lipid metabolism alteration exist. The first one affects the lipid distribution after aerobic exercise by decreasing the concentration of cholesterol ester transport protein in the plasma. Another mechanism explaining HDL alteration is the “modulation of reverse cholesterol transport” [34], a process that removes excess cholesterol from peripheral tissues and sends it to the liver, where it will be eliminated from the body [37]. The modulation of this process is induced by increased expression of the “transcription factor liver X receptor (LXR), and the membrane-associated ATP-binding cassette transporter A-1 (ABCA1) in macrophages” [34]. This could explain the cholesterol-level improvement reported in one study [29]. Regular PE increases HDL-C and compensates for LDL-C and triglycerides. A decrease in LDL-C and triglycerides happens during more intense exercise [38]. Lipoprotein lipase concentration and activity are increased with regular exercise, resulting in higher hydrolysis of triglycerides [34]. However, triglyceride levels did not improve in the study [29]. Another study specifies that even if triglyceride concentration is widely reduced for most of the population, the concentration will increase in 10% of the population due to genetic variances [34]. This and the moderate intensity of the intervention might explain the results found in one of the studies [29]. Glucose exercise-induced variability is affected by the above-described transport and metabolism. During long-term aerobic exercise programs, the GLUT4 protein level is increased, and other glucose transport isoforms are involved, which promote glucose transport. Hexokinase levels and activity are also increased, resulting in increased glucose metabolism, as it is the molecule that phosphorylates glucose during the first step of glycolysis. Other glucose transporters, possibly GLUT1, GLUT3, GLUT6, GLUT10, and/or SGLT3, are involved during strength training. The role of hexokinase is not yet clearly defined in resistance training [39]. During aerobic and eccentric exercise, glucose variability is also influenced by the increased interleukin-6 levels and uric acid. It increases other anti-inflammatory molecules that suppress inflammation [40]. For non-diabetics and diabetics, blood glucose is improved after a brief, high-intensity exercise [41]. The lack of changes in the glucose level in the EG and the increase in the glucose level in the CG [29] might reflect the efficacy of an intervention to regulate blood glucose. Those exercise-induced metabolic changes are important, representing a risk for cardiometabolic disease and mortality [21].

It is important to notice that nutrition was not controlled during the different studies. It can be a factor to consider when interpreting previous results, as it can represent a skew, considering that dietary patterns have an influence on the maintenance of risk factors, including the body mass index, blood pressure, weight, and blood measures. Binge eating, selective food choices, and different production of pro- and anti-inflammatory hormones can lead to obesity and increased fat mass in the population with ASD [42]. Fat mass loss is even more important if dietary caloric restriction complements physical exercise [33]. Cholesterol [43], triglycerides, and glucose balance are also affected by nutrition [44]. Moreover, some specific diets are sometimes used to reduce behavioral and cognitive symptoms linked with ASD [45]. A nutrition program in synergy with physical activity to improve those characteristics might be interesting. The role of diet in managing cardiometabolic health is well established, with strong evidence supporting the benefits of dietary patterns such as the Mediterranean diet and the Dietary Approaches to Stop Hypertension (DASH) diet. These diets emphasize nutrient-dense, minimally processed foods, which contribute to improved cardiovascular and metabolic outcomes. Reducing sodium intake is particularly advantageous for individuals with hypertension, further enhancing the cardioprotective effects of a healthy diet [46]. Therefore, dietary control could further enhance the beneficial impact of exercise on cardiometabolic health.

Three main mechanisms increase the autonomic nervous system function, as reported: “increased parasympathetic nerve activity, decreased sympathetic nerve activity, and the role of the vagal nerve on sympathetic-parasympathetic” [47]. Their meta-analysis concludes that moderate-to-vigorous physical activity enhances parasympathetic nerve activity and inhibits sympathetic nerve activity, ultimately decreasing the HR, as reported in the results of one study [30]. HR improvement might be correlated with the standard decrease that the children go through during their growth. Indeed, one study assessed the changes in the HRV caused by the ageing of healthy subjects and reported an “abrupt change at about the age of 12 years old” [48]. Another study reports that since the mean age of the participants was 11.07 (±1.73), it might be a factor of change in the HR, affecting the validity of the results [30]. Further investigations might be needed to understand the effects of ageing on the HRV in the neuroatypical population. These results are important since lower parasympathetic modulation of the heart with increased sympathetic activation can increase arrhythmia risks and mortality [49].

A study reminds us that VO_2_max might be limited by “the capacity to transport oxygen (e.g., cardiac output), oxygen diffusion to working muscles (e.g., capillary density, membrane permeability, muscle myoglobin content), and adenosine triphosphate (ATP) generation (e.g., mitochondrial density, protein concentrations)” [50]. According to the authors, those variables are improved by exercise. One study declares that endurance training-induced physiologic hypertrophy of the heart’s wall promotes enhanced lusitropy and diastolic left ventricular filling [34]. Eventually, these changes improve the cardiac output and VO_2_max, corroborating the latter’s results [30].

Cardiometabolic health changes seem to be affected by the severity of ASD. All outcomes present statistical differences between mild ASD and severe ASD groups, except for muscle strength, which is similarly changed in both groups [27]. However, when comparing the results of populations with severe ASD [27,28], the changes observed are not similar.

Other factors specific to each individual that can affect the results are gender and age. Indeed, these are important factors in cardiovascular disease (CVD) risk, according to several specific scales assessing this risk, as reported in a previous work [21]. The authors declare that males’ CVD is more likely to happen to males in general, especially if they are ≥45 years old, or to females ≥55 years). In addition, some studies reveal that obese females are more likely to develop cardiometabolic disease than obese males, especially in younger individuals between 35 and 40 years old [51]. Other authors declare that males present higher prevalence of metabolic syndrome (MetS) and CVD than females [52]. The authors describe some factors like advanced age, low-calorie intake, and obesity increasing the risk of CVD for both males and females, and females present additional risk factors, such as higher physical activity. Fat distribution differs in men and women, especially after puberty: women have more body fat and men have a more central fat distribution [53]. This raises an issue of interpretation of the results of the four selected studies, as the male/female ratio is not similar in each study and each group observed. Moreover, it is important to notice that females diagnosed with ASD tend to present fewer autistic symptoms than males with ASD. This could affect the intervention’s compliance and effectiveness if the procedure and the exercises are more easily understood [54]. The effects of exercise are also affected by gender and age: males, persons younger than 50 years old, and persons suffering from hypertension, hyperlipidemia, type 2 diabetes, or MetS seem to obtain better results from exercise interventions [15]. All these factors should be considered when interpreting the results of the four studies retrieved, and their implications emphasize the need for additional investigations.

### 4.2. Secondary Outcomes

The increase in QoL through physical activity observed in one study [29] can be explained by the improvement in the social and functional skills induced by the implementation of a sports program [55]. Population with ASD are at higher risk of mental health problems like depression, obsessive–compulsive disorder, anxiety, and attention deficit hyperactivity disorder [25,56]. Adults perceive the diminution in negative psychological factors as influencing QoL [57]. This is important, since the parents/guardians of the participants assessed the QoL of their child [29]. Another study suggests that exercise induces several mechanisms that might improve depression [58]. The authors report that physical activity causes neuroplasticity, decreases inflammatory factors, protects from oxidative stress and increases antioxidant levels, and affects the neuroendocrine system response in healthy subjects, which are factors correlated with depression. Exercise improves body image and self-efficacy, reduces anxiety, and improves self-esteem. Interactions with others are promoted and eased by physical activity; the authors of [59] report that repetitive behaviors typical of ASD tend to be positively affected. Concerning physical health improvement in the population with mild ASD reported by [27], training-induced skeletal muscle adaptations might be related to changes in contractile and mitochondrial function, contractile protein, intracellular signaling, transcriptional responses, and metabolic regulation [60]. A study explains that a negative energy balance can cause fat mass loss following exercise: the breakdown of fat is more consequent than its storage [33]. Another one reported a slight decrease or an increase in fat mass for participants with mild and severe ASD, respectively [27]. This might be due to the lack of nutrition control or insufficient energy expenditure to compensate for fat storage. Our study participants did not improve their weight but increased their activity level, which could explain the increase in the BMR and calorie expenditure during effort [27,28]. Indeed, energy expenditure and fat-free mass, body surface area, or body weight are significantly and positively related, and basal energy needs are proportional to weight and activity level [61].

### 4.3. Practical Application

While one study promoted group activity with both autistic and neurotypical persons [59], others relate that the population with ASD was more compliant with individual sports [42]. The authors explain that the sports program should include aerobic exercises, flexibility, resistance, and neuromuscular training to achieve the best improvements in body composition and fitness. Metabolic health is mainly improved with moderate–vigorous-intensity aerobic exercise [38,40]. The exercise type should be adapted to the impairments of the participant, the environment should be consistent, and instructions should be clear.

More generally, a study declares that neurotypical individuals present better improvement in body mass, cardiorespiratory fitness, and LDL-C concentration when practicing training combining resistance and endurance, especially in males [62]. They explain that interval training is the best way to improve glucose and triglyceride concentration. Several variables, such as the BMI, WC, the allometric index, HDL-C concentration, and the resting HR, are better after going through training including gathering exercises like “small-sided games in recreational sports, high-intensity functional training, integrated neuromuscular training, cardio resistance training, and multimodal training”, especially in females. Finally, the authors do not recommend endurance training as the first intervention to improve cardiometabolic health. Another study affirms that motor and social functioning improvements occur in all types of exercises [63].

A study provided exercise guidelines specific to the population with ASD; these should, however, be considered cautiously, as the guidelines are based on different types of populations [42]. Considering the heterogeneity of the outcomes of the systematic review, we recommend the adaptation of the frequency, intensity, time, and type (FITT) of exercise to each individual by professionals, considering the different levels of support demanded by people with ASD and compliance of the patient. We advise practitioners to provide a program that gathers muscle strengthening and endurance with moderate–vigorous intensity, for a minimum of two times per week, to obtain the greatest exercise-induced changes in cardiometabolic health.

However, in order to enhance exercise interventions for individuals with ASD, future research should focus on exploring long-term outcomes, evaluating the effects of different exercise modalities, and investigating factors influencing adherence, such as individual preferences and environmental considerations. Practitioners are encouraged to design individualized, enjoyable programs that integrate aerobic, resistance, and neuromuscular exercises, taking into account sensory needs and clear communication. Additionally, involving caregivers and adapting programs to ensure sustainability can improve both physical and social outcomes, while long-term studies are needed to assess the lasting impact on metabolic health and fitness. Moreover, methodological aspects should be addressed in future studies, with an emphasis on sample size calculation, standardized outcome measures, and the protocol of interventions, which could also be considered aspects of tailored treatments.

### 4.4. Strengths and Limitations

To our knowledge, this is the first systematic review that reports the effect of PE on cardiometabolic health in a population with ASD. The outcome of interest presents little available research, with only four studies being appraised. This resulted in the assessment of heterogeneous outcomes, increasing the limitation of this systematic review. The restricted population sample might not represent the whole population, and the results are not homogeneous. Additionally, given that the groups were mixed, there may be an influence on the results, even if women are outnumbered by men in all studies in people with ASD [64].

While the study’s sample primarily consisted of patients with ASD, it also included individuals with other symptoms or conditions. The methodological quality assessment reports a lack of external and internal validity of the results of the included studies and presents selection bias. All these methodological problems highlight the need for further research on this subject. A study reports the results of participants with both mild and severe ASD, which is an interesting direction of research [27]. However, the quality of the study is fair, and the authors did not report if the pre–post-intervention changes were statistically significant. The studies lack consensus on the intervention type, complicating the interpretation of the results. Understanding how to adapt rehabilitation to the patient’s profile for practical application could be interesting. This review’s results should be considered cautiously, as they present some important limitations.

## 5. Conclusions

The results from this systematic review demonstrate that cardiorespiratory fitness, the WHT5, the BMI, cholesterol levels, calorie expenditure, autistic traits scale scores, and QoL are improved after aerobic and functional training in the population with ASD. Moreover, the population with mild ASD improved their BMR, body composition, muscle strength, and endurance after functional training. This systematic review presents the first insights into the effects of PE on cardiometabolic health outcomes in populations with ASD. Although this systematic review serves as an important starting point with promising findings which could be explored in the future, we would like to emphasize the limitation of including a small number of studies, which highlights a gap in the literature regarding physical exercise and its effects on individuals with ASD. Given the lack of studies available, further research is needed to address this topic better and determine the relationship between the severity of ASD and the changes obtained by the implementation of PE.

## Figures and Tables

**Table 1:** Characteristics of the studies and populations.

Study	Groups and Gender (M/F)	Age (Years)	Weight (kg)	Height (cm)	BMI (kg/m^2^)	Waist Circumference (cm)	Cholesterol (Total, HDL-C, and LDL-C) (mg/dL)	Glucose (mg/dL)	Triglycerides (mg/dL)	HR (ppm)	VO_2_max (mL/kg/min)
[28]	EG: n = 5 CG: n = 5 M:6 F:4	14–19 EG: 16.6 ± 1.9 CG: 17.4 ± 1.1	EG: 98.0 ± 18.3 CG: 93.0 ± 32.3	EG: 173.0 ± 7.8 CG: 172.6 ± 8.4	TWG: 33 ± 7.8 CG: 30.9 ± 8.49	NR	NR	NR	NR	NR	NR
[29]	EG: n = 46 CG: n = 18 M: 56 F: 8	6–12	EG:35.9 (13.6) CG: 51.2 (29.1)	EG: 128.2 (14.7) CG: 148.7 (27.2)	EG: 21.4 (6.2) CG: 21.1 (5.4)	EG: 67.9 (16.3) CG: 48.6 (11.1)	Total cholesterol: EG: 167.4 (31.3) CG: 201.0 (49.1) HDL-C: EG:50.3 (14.4) CG: 45.6 (15.5) LDL-C: EG: 100.9 (29.16) CG:119.9 (34.8)	EG: 71.6 (10.2) CG: 89.2 (11.7)	EG: 91.7 (47.0) CG: 139.2 (57.2)	NR	NR
[30]	EG: n = 21 CG: n = 19 M/F: NR	Mean: 11.07 (± 1.73) EG: 11.1 ± 1.9 CG: 11.0 ± 1.5	Mean: 47.71 (± 16.71) EG: 47.7 ± 12.5 CG: 47.6 ± 10.2	Mean: 145.9 (± 15.81) EG: 147.0 ± 15.7 CG: 144.5 ± 15.9	NR	EG: 72.5 ± 7.5 CG: 73.5 ± 4.9 Allometric index (WHT5): EG: 0.60 ± 0.05 CG: 0.61 ± 0.04	NR	NR	NR		EG:73.60 ± 7.0 CG: 72.3 ± 7.8
[27]	Mild group: n = 17, Severe group n = 18 M: 35	20–29 Mild ASD: 22.82 ± 2.70 Severe ASD: 22.83 ± 2.81	Mild ASD: 73.02 ± 12.63 Severe ASD: 72.09 ± 10.32	Mild ASD: 174.01 ± 7.16 Severe ASD: 173.39 ± 8.56	Mild ASD: 24.18 ± 4.32 Severe ASD: 24.14 ± 4.18	NR	NR	NR	NR		NR

TWG: treadmill walking group; EG: experimental group; CG: control group; NR: non-reportable; ASD: autism spectrum disorder; HDL: high-density lipoprotein; LDL: low-density lipoprotein.

**Table 2:** Characteristics of interventions.

Study	Type of Exercise and Duration of Intervention	Frequency	Protocol	Training Sessions (reps or min)	Control Group Intervention
[28]	Treadmill walking 9 months (39 weeks)	The initial frequency was 2 times/wk Progression of 1 day every 2 weeks Peak frequency of 5 times/wk	Initial speed: 2.4–3.5 mph (according to individual capacity) Progression: 0.1–0.3 mph every 2–3 weeks Peak: 2.7–4.1 mph	Initial: 8 min Progression: 1–2 min every 2–3 weeks Peak: 20 min	‘Leisure activity’ 30 min, 3 times/wk
[29]	Coordination and strength exercise 48 weeks (11 months)	2 times/wk	Climbing and support on the bar: 5.0 s Release to the basket: different weights (0.5, 1.0, and 2.0 kg) Work with elastics: NR Walking on steps and inclined plane: three steps (12 and 15 cm) and inclined plane of 0.78 cm in length and 30 cm Step box with target: three sets of sequenced steps of dimension 60 × 28 × 14 cm Sequenced march: running on a sequence of five arcs	40 min 5 min of preparation 30 min of warm-up and exercise 5 min of return to calm state (relaxation)	Usual levels of daily activity + standardized care
[30]	Judo program supervised by judo instructors, 6 months (26 weeks)	1/wk	Different types of movements and falling techniques: From ‘walking in all directions’ to ‘change in direction’ activities, from stable movements to unstable movements. Judo analytical techniques and judo games: Progressively increasing body contact with games, simplifying movements to focus on essential judo movements. Ground control techniques and throws: Incremental change to already known movements, progression from repetitive movements to those more relevant to the understanding and purpose of judo Repetitions of basic movements in different directions and planes (pulling, pushing, holding, and lifting).	90 min sessions 15–20 min (movement and falling techniques) 25–30 min (judo games) 25–30 min (ground control techniques and throws) 20–30 min (basic movement)	No extracurricular sports activities
[27]	Supervised educational exercise program, 12 weeks (2.7 months)	2 times/wk	From week 1 to 4: Game Catching a tail, 15 min Playing tug-of-war, 15 min Floorball Play for passing and receiving a ball, 15 min Passing a ball (slapper and air pass), 15 min. From week 5 to 8: Floorball Mini-short game, 15 min Basketball Dribble (a hand dribble and then both-hands dribble), 15 min Pass (chest, overhand, one hand, and bound), 15 min Shot (middle, layup, and pass and shoot), 15 min Inline skating Skating adaptation (wearing, falling, and standing), 10 min Walking with an expert, 5 min. From week 9 to 12: Inline skating Pushing (kneeling and left or right foot push), 15 min Turn (return to target and turn left/right/front/back), 15 min Jumping play Sprint, jump over obstacles, running, and jump, 20 min High jump and triple jump, 20 min.	5 min of warm-up 40 to 45 min of workout 10 min of stretching	NR (no control group)

wk: week; min: minutes, NR: not reported.

**Table 3:** Effect of the interventions on the outcomes.

Study	Weight (kg)	BMI (kg/m^2^)	Waist Circumference (cm)	Cholesterol (Total, HDL-C and LDL-C (mg/dL)	Glucose (mg/dL)	Triglycerides (mg/dL)	HR_baseline_ (ppm)	VO_2_max (ML/kg/min)	Secondary Outcomes
[28]	EG: non-significant reduction in weight, 92.9 ± 15.5 *p* = 0.065. CG: non-significant reduction in weight 91.2 ± 30.9, *p* = 0.215.	EG: a significant decrease, 30.2 ± 6.33, *p* = 0.016. CG: no significant changes, 30.0 ± 7.35, *p* = 0.315.	NR	NR	NR	NR	NR	NR	Calorie expenditure Average at month 9: 914.62 ± 233.4 mean ± S.D. increase in calorie expenditure for EG participants.
[29]	No significant changes. EG: −1.9 [0.9; 4.7]. CG: −0.2 [2.1; 2.6].	No significant changes. EG: −1.9 [0.9; 4.7]. CG: −0.2 [2.1; 2.6].	EG: no significant changes, +0.4 [3.6; 4.5]. CG: increased by +1.8 [1.6; 5.2].	EG: increased HDL-C, + 5.2 [2.2; 8.1]. CG: increased HDL-C + 0.9 [−1.6; 3.4]. EG: decreased LDL-C, −7.7 [−14.5; −0.9]. CG: increased LDL-C, + 2 [−3.8; 7.8]. EG: decreased total cholesterol, −10.1 [−19.0; 1.3]. CG: increased total cholesterol + 0.2 [−7.2; 7.7].	EG: no significant changes, −1.5 [4.7; 1.6]. CG: increase of +2.8 [0.2; 5.5].	EG: no significant change, + 33.1 [−8.6; 74.9]. CG: decreased by −38.1 [−73.5; −2.72].	NR	NR	Autistic traits scale: EG: decreased by −8.1 [−12.2; −4]. CG: decreased by −1.4 [−4.9; 2.1]. Motor profile scale: EG: decreased by −2.4 [−3.3; −1.5]. CG: negligible change of 0.1 [−0.7; 0.9]. Physical health: EG: increased by 13.3 [7.7; 18.9]. CG: increased by 3.1 [−1.8; 8.1]. Psychosocial health: EG: increased by 15.2 [9.8; 20.7]. CG: decreased by −2.4 [−7.1; 2.4].
[30]	NR	NR	EG: decreased WC by −1.86 [−2.63; −1.09]. CG: negligible change of +0.32 [−0.49; 1.23]. Allometric index (WHT5): Decreased in both EG and CG. EG has a very large decrease, −0.024 [−0.030; −0.019]. CG: medium decrease, −0.008 [−0.015; −0.002].	NR	NR	NR	Decreased in EG and CG. EG: medium decrease of 68.3 ± 4.4, *p* < 0.001, Apost–pre = 28.0%. CG: negligible decrease of 70.6 ± 5.5, *p* = 0.018, Apost–pre = 45.6%.	EG: medium increase of 55.2 ± 7.5, *p* < 0.001, Apost–pre = 66.6%; CG: no significant changes, 54.2 ± 6.2; *p* = 0.609, Apost–pre = 51.4%.	NR
[27]	Mild ASD: decreased ~2.34%. Severe ASD: increased ~1.70%.	Mild ASD: decreased by ~2.34%. Severe ASD: increased ~1.70%.	NR	NR	NR	NR	NR	NR	Shuttle run test (s): Mild ASD increased by ~8.49%. Severe ASD: decreased by ~2.88%. Fat mass (kg): Mild ASD: decreased by ~0.28%. Severe ASD: increased by ~5.60%. Strength (kg): Mild ASD: increased by ~16.92%. Severe ASD: increased by ~17.20%. Muscle endurance (rep): Mild ASD: increased by ~11.34%. Severe ASD: decreased by ~12.48%. Basal metabolic rate: Mild ASD: increased by ~0.75%. severe ASD: decreased by ~1.39%.

EG: experimental group; CG: control group; NR: non-reportable; ASD: autism spectrum disorder; HDL: high-density lipoprotein; LDL: low-density lipoprotein; 95%; Apost–pre: “stochastic superiority, which represents the probability that a randomly selected score from the post-intervention will be greater than a randomly selected score from the pre-intervention”.

**Table 4:** Downs and Black quality assessment.

First Author, Year	1	2	3	4	5	6	7	8	9	10	11	12	13	14	15	16	17	18	19	20	21	22	23	24	25	26	27	Total	Quality
[28]	1	1	1	1	1	1	1	0	1	1	0	0	0	0	0	1	1	1	1	1	1	0	0	0	0	1	0	16/28	Fair
[29]	1	1	1	1	2	1	1	1	1	1	0	0	1	0	1	1	1	1	1	1	1	0	1	0	0	0	1	21/28	Good
[30]	1	1	1	1	0	1	1	0	0	1	0	0	0	0	0	1	1	1	1	1	1	0	0	0	0	0	1	14/28	Poor
[27]	1	1	1	1	0	1	1	0	1	1	0	0	0	1	0	1	1	1	1	1	0	0	0	0	0	0	1	15/28	Fair

Note: Description of Downs and Black items can be found in [18]. Quality of evidence was grouped as excellent (26–28), good (20–25), fair (15–19), and poor (≤14).

## Data Availability

No new data were created or analyzed in this study. Data sharing is not applicable to this article.
